# Evolutionary Relationships Between Low Potential Ferredoxin and Flavodoxin Electron Carriers

**DOI:** 10.3389/fenrg.2019.00079

**Published:** 2019-08-23

**Authors:** Ian J. Campbell, George N. Bennett, Jonathan J. Silberg

**Affiliations:** 1 Biochemistry and Cell Biology Graduate Program, Rice University, Houston, TX, United States; 2 Department of BioSciences, Rice University, Houston, TX, United States; 3 Department of Chemical and Biomolecular Engineering, Rice University, Houston, TX, United States; 4 Department of Bioengineering, Rice University Houston, TX, United States

**Keywords:** electron transfer, evolution, ferredoxin, flavin mononucleotide, flavodoxin, iron-sulfur cluster, oxidoreductase, oxidative stress

## Abstract

Proteins from the ferredoxin (Fd) and flavodoxin (Fld) families function as low potential electrical transfer hubs in cells, at times mediating electron transfer between overlapping sets of oxidoreductases. To better understand protein electron carrier (PEC) use across the domains of life, we evaluated the distribution of genes encoding [4Fe-4S] Fd, [2Fe-2S] Fd, and Fld electron carriers in over 7,000 organisms. Our analysis targeted genes encoding small PEC genes encoding proteins having ≤200 residues. We find that the average number of small PEC genes per Archaea (~13), Bacteria (~8), and Eukarya (~3) genome varies, with some organisms containing as many as 54 total PEC genes. Organisms fall into three groups, including those lacking genes encoding low potential PECs (3%), specialists with a single PEC gene type (20%), and generalists that utilize multiple PEC types (77%). Mapping PEC gene usage onto an evolutionary tree highlights the prevalence of [4Fe-4S] Fds in ancient organisms that are deeply rooted, the expansion of [2Fe-2S] Fds with the advent of photosynthesis and a concomitant decrease in [4Fe-4S] Fds, and the expansion of Flds in organisms that inhabit low-iron host environments. Surprisingly, [4Fe-4S] Fds present a similar abundance in aerobes as [2Fe-2S] Fds. This bioinformatic study highlights understudied PECs whose structure, stability, and partner specificity should be further characterized.

## INTRODUCTION

Redox-active cofactors are essential components of metabolism, functioning as molecules that transfer electrons at various reduction potentials, according to metabolic need. These pools of small molecules (e.g., NADH, NADPH, FADH, FMN, riboflavin, and quinones) can couple their reducing power to a wide range of oxidoreductases in parallel. For example, the quinone that functions in aerobic respiration within *Escherichia coli* interacts with two dozen oxidoreductases, while NADH/NADPH are used by over one hundred oxidoreductases in this microbe (Orth et al., 2011). What these small molecules lack, however, is the ability to evolve structures that discriminate partner binding and tune their midpoint potentials. In contrast, protein electron carriers (PECs) can tune both reduction potential and partner binding by modifying their amino acid sequences (Hosseinzadeh and Lu, 2016; Kim et al., 2016). This tunability is thought to enable the evolution of protein-controlled, energy-conserving electron transfer (ET) pathways.

The Iron-Sulfur World Hypothesis, that life evolved within the cavities and capillaries of iron-sulfur enriched mounds, implicates ferredoxins (Fds) with [4Fe-4S] clusters as the earliest low potential PECs (Sousa et al., 2013). This idea is supported by the observations that these proteins represent the smallest PECs, having as few as 55 amino acids (Bertini et al., 1995), and the observation that iron and sulfur can readily combine to form iron-sulfur clusters under anaerobic conditions (Venkateswara Rao and Holm, 2004). With the advent of oxygenic photosynthesis and the Great Oxidation Event, the usage of O_2_-sensitive [4Fe-4S] Fds may have been disincentivized relative to the more O_2_-tolerant [2Fe-2S] Fds. Support for this idea comes from the observation that [4Fe-4S] clusters within canonical bacterial Fds have an exposed sulfido atom which can be attacked by O_2_, resulting in the release of iron and destruction of the cluster (Imlay, 2006; Jagannathan and Golbeck, 2009). With a hydrophobic patch covering their metallocluster, [2Fe-2S] Fds are more shielded from this degradation (Pierella Karlusich et al., 2014), while still capable of presenting a similar range of midpoint potentials (Atkinson et al., 2016). As a result, [2Fe-2S] Fds are thought to have emerged as the favored electron acceptor for the O_2_-evolving photosystem, proliferating in the new O_2_-rich world. While it is clear that some [4Fe-4S] Fds are more sensitive to O_2_ than [2Fe-2S] Fds, the extent to which these two Fd types are used across different environmental niches has not been well established.

The rise in global O_2_ concentration created insoluble iron hydroxides, thereby lowering the amount of bioavailable iron (Ilbert and Bonnefoy, 2013; Pierella Karlusich et al., 2014). It was at this time that flavodoxins (Flds) are thought to have risen in popularity, especially as an iron limitation response for phytoplankton (Pierella Karlusich et al., 2014). Flds use flavin mononucleotide (FMN) as their redox cofactor and are able to substitute for [2Fe-2S] Fds in a wide range of ET reactions within phototrophs (Demuez et al., 2007; Goñi et al., 2008; Pierella Karlusich et al., 2014). Despite an increasing body of Fld research (Freigang et al., 2002; Demuez et al., 2007; Goñi et al., 2008; Pierella Karlusich et al., 2014; Mellor et al., 2017), we still do not know how prevalent Flds are across extant organisms and how they work with Fds to manage ET across different biochemical pathways.

Gene duplication events during evolution have led to the growth and diversification of PECs (Onda et al., 2000; Terauchi et al., 2009; Peden et al., 2013; Cassier-Chauvat and Chauvat, 2014; Pierella Karlusich et al., 2014; Atkinson et al., 2016; Mellor et al., 2017; Burkhart et al., 2019). Biochemical and cellular studies of [2Fe-2S] Fd paralog specialization have been performed in a hyperthermophilic Archaeon, *Thermococcus kodakarensis* (Burkhart et al., 2019), and three model photosynthetic organisms: *Synechocystic* sp. PCC6803, *Zea mays*, and *Chlamydomonas reinhardtii* (Onda et al., 2000; Terauchi et al., 2009; Cassier-Chauvat and Chauvat, 2014). These studies revealed variations in the pattern of expression as well as differences in partner binding. However, we still lack basic information on how these and other low potential PECs evolved and specialized across the tree of life. While we have some limited information on the number of Flds and [4Fe-4S] Fds in phototrophs, beyond these organisms estimates of PEC distribution are scarce. We know that Flds often replace [2Fe-2S] Fds under iron limited conditions, both in free-living organisms and host-associated organisms (Freigang et al., 2002; Goñi et al., 2008), but a quantitative description at the genomic level is lacking.

To gain a better understanding of how evolution has selected extant PECs, we report on the genome mining of 7,079 organisms for sequence motifs that are characteristic of three different low-potential PEC families, including the [4Fe-4S] Fds, [2Fe-2S] Fds, and Flds. We show that many organisms have large pools of small PEC genes, with 50% of our analyzed organisms possessing six or more small PEC genes, including members of all three PEC types. We find that PEC pools vary across genomes, with some organisms lacking small PEC genes and others having >50 total small PEC genes. We also report that PEC genes elongate in organisms having multiple PEC-encoding genes and under certain environmental conditions.

## MATERIALS AND METHODS

### Genome Mining

We harvested 7079 genomes from the Joint Genome Institute (JGI) with the “finished” sequencing status and scanned them for genes matching Interpro sequence signatures for Fld/nitric oxide synthase (IPR008254), [2Fe-2S] Fd-cluster binding domain (IPR001041), and [4Fe-4S] Fd-cluster binding domain (IPR017896) (Finn et al., 2017). Interpro annotation was chosen because it synthesizes signatures from multiple databases with complementary but distinct annotation strategies, such as Pfam and PROSITE, which match for proteins on the basis of Hidden Markov Models and shared alignment profiles, respectively (Finn et al., 2017). Genes with over 200 amino acids were excluded from the dataset to focus the analysis on small PECs. Metadata for each genome was also downloaded and used for environmental niche analysis. Sequences obtained from genome-mining were collected in a FASTA format and analyzed for Interpro (IPR) sequence signatures with InterProScan 5 (Jones et al., 2014). Supplementary Dataset 1 contains a list of organisms with the number of genes having each IPR signature and metadata about ecosystem characteristics. It also contains a list of the individual PECs collected after genome mining, with their sequences and the metadata of their host organisms.

### Analysis of PEC Structures

The protein data bank was used to compile structures of [4Fe-4S] Fds, [2Fe-2S] Fds, and Flds (Berman, 2000). For [4Fe-4S] Fds, we used PDB identifiers (IDs) 1DUR, 2FDN, 1CLF, 1FXD, 1VJW, 1FXR, 1SJ1, 4ID8, 1BC6, 1BWE, 1H98, 4KT0, 1RGV, 1JB0, 1IQZ, 1BLU, 2FGO, 2ZVS, 2VKR, 1XER, 2V2K, and 7FDR. For [2Fe-2S] Fds, we used PDB IDs 1L5P, 4ITK, 1WRI, 1AWD, 2MH7, 1FRR, 4ZHO, 3AV8, 1PFD, 1OFF, 5H57, 3AB5, 1ROE, 1A70, 4FXC, 3B2G, 3B2F, 1RFK, 1JQ4, 1IUE, 1FRD, 1CZP, 2WLB, 3LXF, 1B9R, 1UWM, 1PDX, 1M2D, 1I7H, 3AH7, 2MJD, 2MJ3, 2Y5C, 5FFI, 3P1M, 1E0Z, 1DOI, and 1AYF. For Flds, we used PDB IDs 2FZ5, 1FLD, 4HEQ, 2HNA, 2FX2, 3F6R, 3KAP, 5B3L, 4OXX, 1FUE, 2BMV, 2W5U, 1CZH, 1FLV, 1OBO, 2MT9, 2FCR, 1AG9, 1YOB, 2WC1, and 2ARK.

### Pairwise Analysis of PEC Abundance

The gene counts for each PEC type were plotted against one another in heatmaps generated using the Matplotlib python package (Hunter, 2007). A log-scaled color gradient was used to illustrate the number of organism with each PEC count combination. For ternary plot analysis, organisms were binned on the basis of their relative PEC pool composition. For each organism, counts of each PEC family were normalized such that they summed to 100%. All combinations of PEC pools were divided into bins with step sizes of 10%. If an organism’s PEC composition placed it at the boundary of two or more bins, it was randomly placed in one of the adjoining bins. The density of organisms in each bin were visualized using a viridis color gradient. Results were plotted using the python-ternary package (Harper et al., 2015).

### PEC Phylogeny Mapping

Evolutionary analysis was performed using a previously described evolutionary tree derived from concatenated protein sequences (Hug et al., 2016). The tree was pruned down to the 351 organisms present in our dataset using the Environment for Tree Exploration (ETE) 3 python toolkit (Huerta-Cepas et al., 2016). The IPR-matching PEC gene counts were placed at each organisms leaf in the phylogenetic tree in a stacked bar graph. Data was visualized using the Interactive Tree of Life webservice (Letunic and Bork, 2016).

### Environmental Niche Analysis

Organisms were sorted on the basis of their JGI metadata labels. Organisms labeled thermotolerant were placed into the thermophile bins, while those labeled psychrotolerant and psychrotrophic were placed in the psychrophile bin. A heatmap was generated by plotting the average PEC gene count for organisms at each O_2_ niche and growth temperature bin. Average values were rounded to two significant figures. Average PEC counts for external pH values were calculated by dividing PEC counts by the number of organisms observed at each pH.

### PEC Length Analysis

To generate plots showing PEC length distributions and abundances, organisms were divided into different categories (PEC gene count, O_2_ requirement, growth temperature), and the length distributions in each category were smoothed using kernel density estimation via the Matplotlib package (Hunter, 2007).

### Statistics

The weighted pH average of each PEC type was calculated, along with the weighted standard deviation, and the three distributions were compared using a paired, two-tail *t*-test calculated using NumPy (van der Walt et al., 2011).

## RESULTS

### Genome-Mining Strategy

To understand how low-potential PEC usage varies in nature, we downloaded 7079 genomes with the sequencing status “Finished” from the Integrated Microbial Genomes and Microbiomes database (Chen et al., 2019). A majority of the genomes were from Bacteria (*n* = 6,733), although Archaea (*n* = 275) and Eukaryotes (*n* = 71) were represented. We scanned each genome for genes having Fld, [2Fe-2S] Fd, and [4Fe-4S] Fd Interpro sequence signatures (Finn et al., 2017). We excluded all gene matches encoding proteins above 200 amino acids. This size cutoff was chosen because it is greater than the largest [4Fe-4S] Fd (106 residues: 7FDR), [2Fe-2S] Fds (128 residues: 1E0Z), and Flds (184 residues: 2ARK) reported in the PDB (Schipke et al., 1999; Marg et al., 2005). By using a size cutoff that exceeds the length of PECs with single domain structures in the PDB, we sought to evaluate how the size of these three PEC families varies beyond the family members that have already been studied. We applied the same size cutoff to all three PEC types, even though they differ in average size within the PDB. Their average sizes are 77 ([4Fe-4S] Fds), 111 ([2Fe-2S] Fds), and 160 (Flds) residues. By using this approach, our analysis allowed the comparison of PEC evolution across the same size range. Additionally, the use of a 200 amino acid cutoff minimized Fld false positives, as IPR008254 also identifies nitric oxide synthases, enzymes that can be >400 amino acids (Fischmann et al., 1999; Fedorov et al., 2003). Because sequence additions frequently do not abolish the function of the ancestral domain (Björklund et al., 2005), we posited that many of the gene sequences detected with our approach encode proteins capable of ET like known Fds and Flds.

### Organisms Differ in Their PEC Gene Counts

**FIGURE 1 |:**
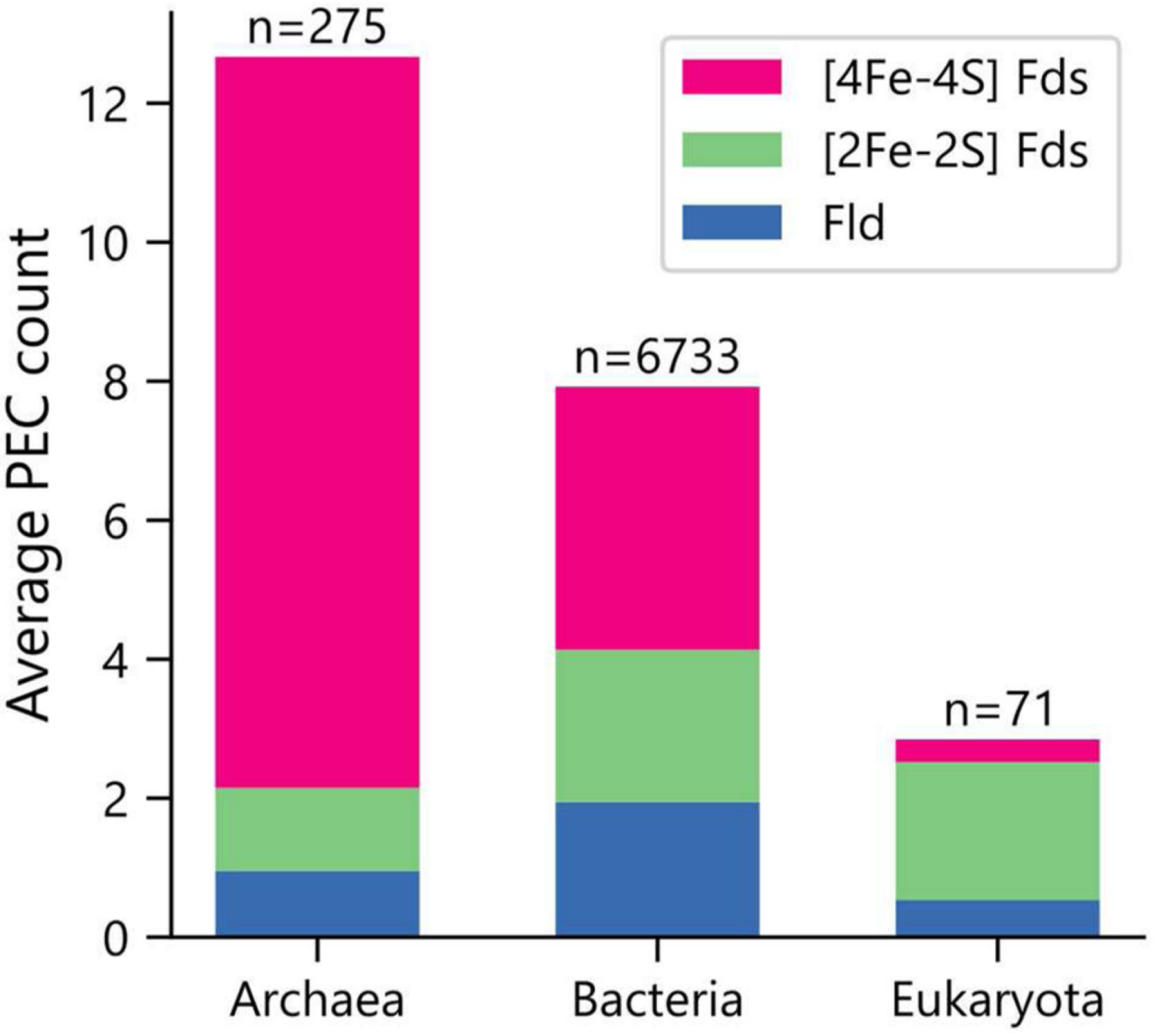
Average PEC gene counts across the domains of life. The average number of PEC genes containing motifs that are characteristic of [4Fe-4S] Fds (red), [2Fe-2S] Fds (green), and Flds (blue) in each domain. The number of genomes analyzed within each domain is shown on top of each bar.

Organisms were initially sorted by taxonomic domain to visualize how gene abundances vary. Figure 1 shows that Archaea have the largest average number of PEC genes per organism (μ = 12.7 ± 5.7), heavily favoring genes with [4Fe-4S] Fd IPR signatures.This observation is in line with previous observations that archaeal metabolisms depend more heavily on non-heme iron-binding proteins than those of the other two domains (Andreini et al., 2007). Eukaryotes present the smallest number of PEC genes per organism (μ = 2.8 ± 5.3), utilizing genes with [2Fe-2S] Fd signatures to the greatest extent. Bacteria have an intermediate level of PEC genes (μ = 7.9 ± 6.2) and favor a more balanced composition.

**FIGURE 2 |:**
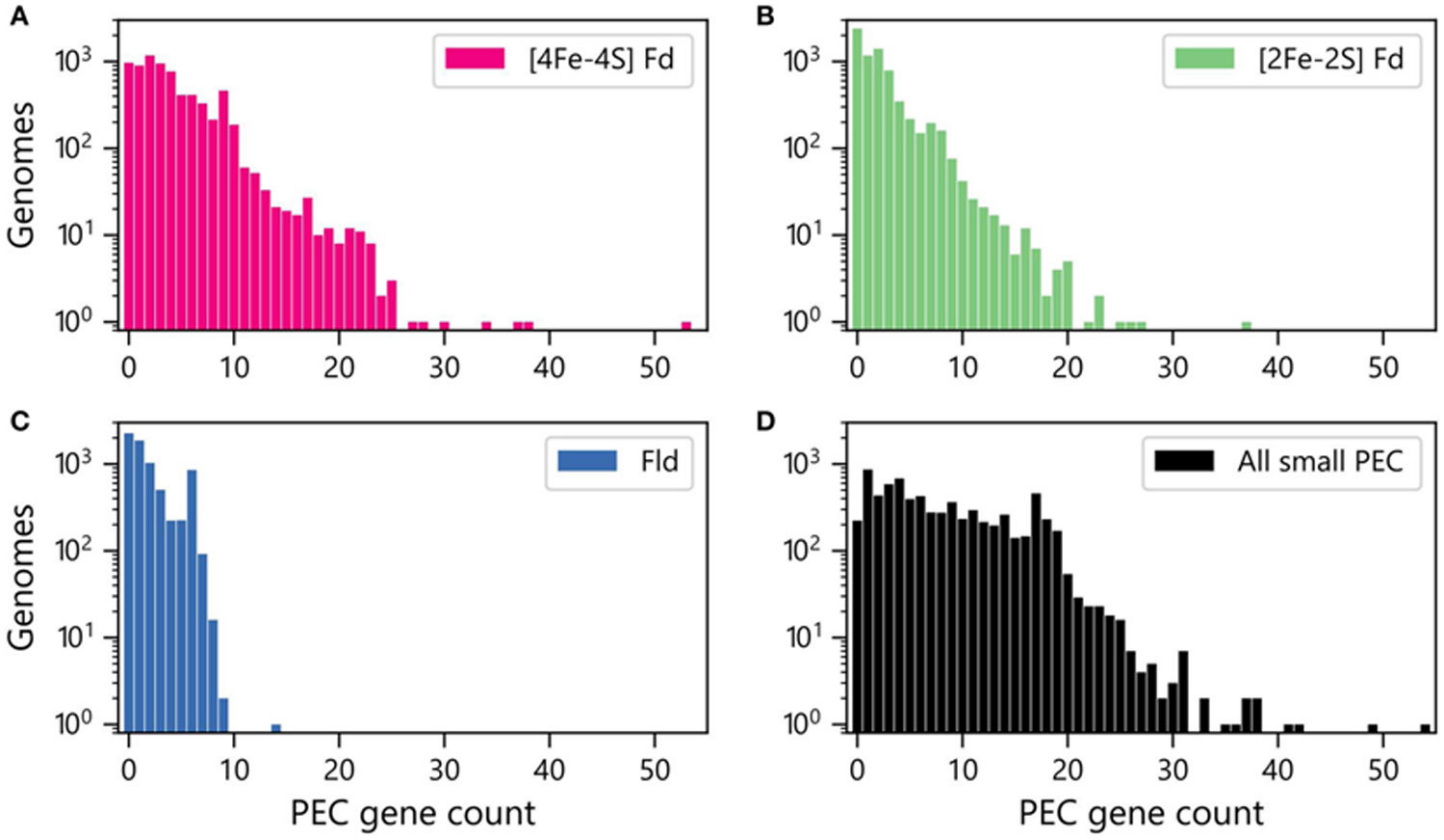
Numbers of genomes having different abundances of PEC genes. Number of organisms with different **(A)** [4Fe-4S] Fd (red), **(B)** [2Fe-2S] Fd (green), and **(C)** Fld (blue) gene counts. **(D)** Number of organisms with different total numbers of PEC genes (black). The data shown was obtained by summing up the Fld, [2Fe-2S] Fd, and [4Fe-4S] Fd counts in each genome.

We next quantified the abundances of genes encoding each PEC type within each genome. Figure 2A shows that [4Fe-4S] Fd genes occur with the greatest abundances, with more than 400 organisms having ten or more [4Fe-4S] Fd genes. In contrast, there are only 162 organisms with 10 or more [2Fe-2S] Fd genes (Figure 2B), and only one organism having more than 10 Fld genes (Figure 2C). The total counts of genes encoding all three PECs were also summed together for each organism (Figure 2D). This analysis reveals that 50% of all organisms analyzed have 6 or more PEC genes, and 3% of the organisms lack small PEC genes.

**FIGURE 3 |:**
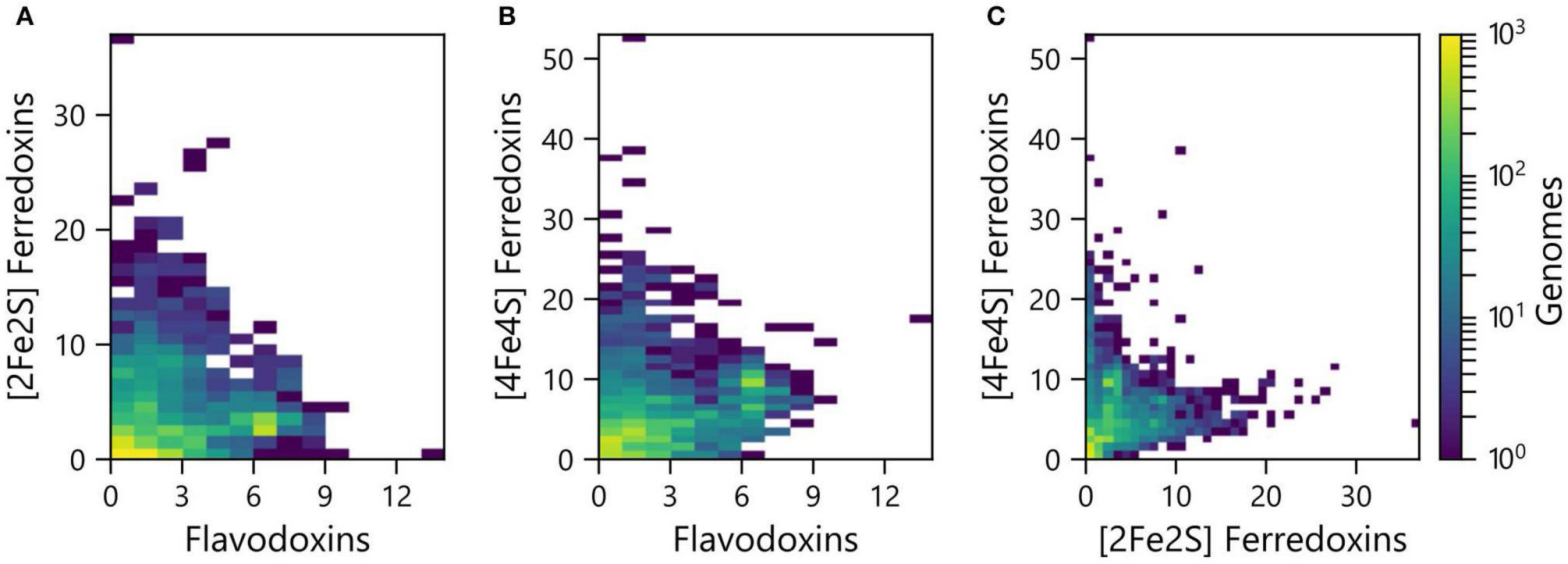
Pairwise abundance of PEC genes within each genome. The abundances of **(A)** [2Fe-2S] Fd and Fld, **(B)** [4Fe-4S] Fd and Fld, and **(C)** [4Fe-4S] Fd and [2Fe-2S] Fd genes are plotted as heat maps. The density of organisms having each pairwise count is shown using a viridis color gradient.

PECs can at times transfer electrons to overlapping sets of partner proteins, suggesting that some have evolved as interchangeable ET hubs. In some organisms, Fds are used for ET under iron-rich conditions, while Flds support ET among the same partner oxidoreductases under limiting iron conditions (Demuez et al., 2007; Goñi et al., 2008; Pierella Karlusich et al., 2014). To evaluate if the abundances of the different PEC gene types are proportional in some organisms or if there are differences in the relative abundances, we compared the pairwise counts of the different PEC types within each genome. All three pairwise comparisons presented a wide range of abundance combinations. In the case of the Fld and [2Fe-2S] Fd comparison (Figure 3A), most organisms have <4 genes encoding each PEC type (64%). A small fraction of organisms (3%) contain ≥4 genes encoding both PEC types, and the remainder have ≥4 of one PEC type and <4 of the other type (33%).

**FIGURE 4 |:**
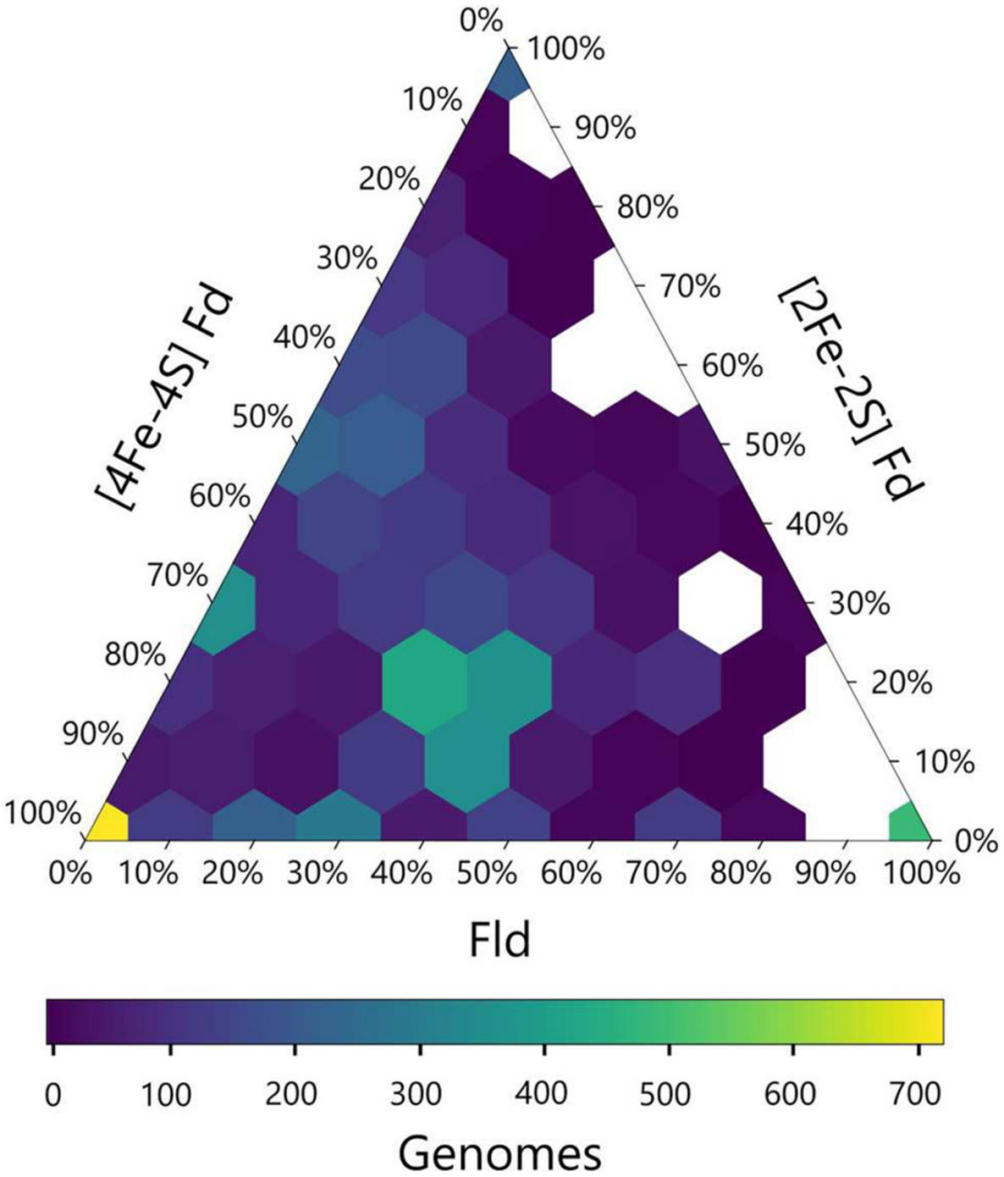
Relative abundances of PEC specialist and generalists. The relative percentage of PEC gene counts for all three families are plotted with the density of organisms at each coordinate illustrated by a viridis color gradient. White hexagons represent combinations that were not observed.

The pairwise relationships between [4Fe-4S] Fds and Flds (Figure 3B) and [4Fe-4S] and [2Fe-2S] Fds (Figure 3C) presented larger number of organisms with higher abundances of both PEC types. With the [4Fe-4S] Fd and Fld comparison, 17% of the organisms have ≥4 of both PEC types, while the [4Fe-4S] and [2Fe-2S] Fd comparison revealed that 12% of the organisms have ≥4 of each PEC type. With these comparisons, the organisms with the highest [4Fe-4S] Fd gene abundances frequently present three or fewer Fld or [2Fe-2S] Fd genes. Of the genomes analyzed, 26% of genomes possess ≥4 [4Fe-4S] Fd genes and <4 Fld genes, while the reverse composition is only observed in 3% of genomes. In addition, 31% possess ≥4 [4Fe-4S] Fd genes and <4 [2Fe-2S] Fd genes, while the reverse composition is only observed in 6% of genomes. These trends support the idea that [4Fe-4S] Fds arose early in evolution and diversified through gene duplications, gaining critical metabolic functions before the widespread use of the [2Fe-2S] Fds and Flds.

We next calculated the abundance of genes encoding all three PEC types in each genome and visualized this data using a ternary plot (Figure 4). The values obtained for each organism were distributed into bins based on the relative percentages of each gene type. With this analysis, ~20% of the organisms had only one type of PEC gene, a small subset lacked all three PEC types (3%), and the remainder of the organisms had two or three PEC types encoded in their genomes (77%). One of the most popular PEC gene pool makeups is near the middle of the ternary plot, representing organisms with 40–50% [4Fe-4S] Fd, 10–20% [2Fe-2S] Fd, and 30–40% Fld genes. This hotspot is attributed to the high number of proteobacteria in our data set, which dominate this makeup.

The ternary plot also reveals PEC combinations that are absent from the dataset organisms. For organisms that lack [4Fe-4S] Fd genes, only a subset of the possible [2Fe-2S] Fd and Fld gene mixtures are observed. Under this constraint, Fld and [2Fe-2S] Fd gene specialists are most common. This trend is consistent with the pairwise PEC comparisons that show organisms only possess large numbers of [2Fe-2S] Fd or Fld genes in cases where they also possess multiple [4Fe-4S] Fd genes. Taken together, these results indicate that genes encoding [4Fe-4S] Fds are widespread with a high degree of penetration in many genomes and provide further evidence that ancient organisms were likely dependent upon the [4Fe-4S] Fds family before the expansion of the other two families.

### PEC Gene Evolution

**FIGURE 5 |:**
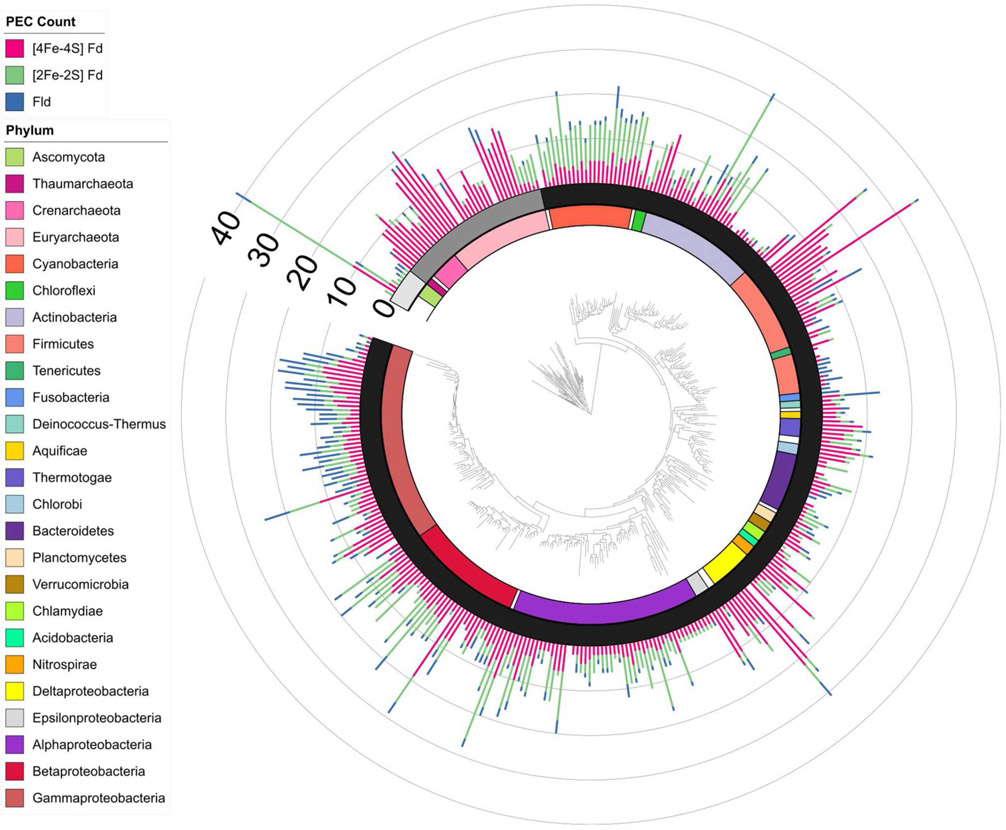
PEC gene abundance mapped onto an evolutionary tree. Bars are used to illustrate the total PEC gene counts of the three different PEC families at the leaves in a stacked bar graph, including [4Fe-4S] Fds (red), [2Fe-2S] Fds (green), and Flds (blue). The domain for each organism is depicted by shading internal to the stacked bars: Eukaryotes (Light gray), Archaea (dark gray), and Bacteria (black). The major phyla and classes of organisms represented in the tree are visualized internal to the domains using the colored bars as noted in the key. Phyla with only one representative are labeled with white bars.

To understand how PEC gene counts vary across extant organisms, we mapped our data onto an evolutionary tree (Hug et al., 2016). For visualization, we pruned the phylogenetic tree down to those organisms represented in our data set (*n* = 351). We show the abundance for all 351 organisms in a bar graph at each of the leaves on a tree (Figure 5) and the average abundance in the major taxonomic groups (Table 1). From this analysis, it appears that evolution has selected for the expansion of homologs of all three PEC types more than once. The [4Fe-4S] Fds are most widespread in overall abundance, being dispersed through most organisms. However, this PEC type clearly spikes in average abundance (>10 paralogs) within the Archaea, Firmicutes, Deltaproteobacteria, and Betaproteobacteria. The [2Fe-2S] Fds present the greatest abundances within phototrophs (plants, Cyanobacteria), Alphaproteobacteria, and Betaproteobacteria. The Flds are most abundant within a subset of the Firmicutes (Bacilli), Gammaproteobacteria, and in parasitic organisms. Below, we describe the trends in greater detail for individual taxonomic groups that are well represented in our data set.

### Eukaryota

In our data set, this kingdom has the smallest number of genomes, including algae, a protozoan, a plant, fungi, and *Homo sapiens*. These organisms exhibit highly variable PEC compositions, although they all possess [2Fe-2S] Fds. Corn (*Zea mays*) possesses the largest number of PECs of any organism on the tree (*n* = 42) and the fourth most PEC genes of any organism in the dataset, utilizing a mix of all three types. This observation is consistent with an ancient plant duplication event and a high rate of retention of duplicate genes in plant genomes (Panchy et al., 2016). With the exception of the green algae *Ostreococcus lucimarinus*, which contains 11 PEC genes, all other organisms in this domain possess small numbers of PEC genes. *Homo sapiens* uses two [2Fe-2S] Fds (Sheftel et al., 2010), while fungi have incorporated one [2Fe-2S] Fd and up to two Flds. Both human Fds are nuclear-encoded and translocated to mitochondria (Sheftel et al., 2010). Additionally, the chloroplasts in phototrophs are hotbeds of PECs (Pierella Karlusich and Carrillo, 2017). Taken together, these observations support the idea that some organisms compartmentalize PECs within organelles that function in light harvesting and energy production (Sheftel et al., 2010; Scheibe and Dietz, 2012).

### Archaea

This domain has the highest average number of PEC genes per genome. The [4Fe-4S] Fds are the most popular PEC type in most Archaea represented. The exception to this trend is the Haloarchaea, which predominately uses [2Fe-2S] Fds. This class is noted for having many members that live aerobically and in extreme saline conditions (Sorokin et al., 2017). This observation is interesting because Haloarchaea are thought to have acquired >1,000 genes from methanogens in a single gene transfer event, and because methanogens are generally anaerobic and dominated by [4Fe-4S] Fds. It seems unlikely that Haloarchaea received large numbers of [2Fe-2S] Fds from this gene transfer event, although they received other oxidoreductases from methanogens, such as pyruvate:Fd oxidoreductase (Nelson-Sathi et al., 2012). Haloarchaea possess [2Fe-2S] Fds with a high-degree of similarity to plant-type Fds, with the exception of a ~30 amino acid addition near the N-terminus, which has been hypothesized to have entered the organism through gene transfer from cyanobacteria (Pfeifer et al., 1993; Marg et al., 2005; Nelson-Sathi et al., 2012). Surprisingly, a few archaea lack genes with Fd and Fld IPR signatures. Two organisms in the phylogenetic tree (*Archaeoglobus fulgidus* DSM 8774 and *Palaeococcus pacificus* DY20341) lack PEC genes, although other organisms from their respective classes that are present in the genome mining dataset contain PEC encoding genes (Supplementary Dataset 1).

### Cyanobacteria

The PEC gene distribution in these organisms most closely matches the Haloarchaea, with averages of 8.6 [2Fe-2S] Fd, 4.5 [4Fe-4S] Fd, and 1.0 Fld genes per genome. This trend makes this one of the richest phyla in terms of consistent, uniform abundance across all three PEC types. This representation is thought to arise in part because Cyanobacteria express Flds instead of [2Fe-2S] Fds under iron-limiting conditions (Pierella Karlusich et al., 2014). Rather than contributing to management ET in chloroplasts, the [4Fe-4S] Fds in these organisms have been implicated in oxidative and metal stress response pathways (Cassier-Chauvat and Chauvat, 2014).

### Chloroflexi

Some of these organisms have genes encoding all three PEC types like Cyanobacteria. However, this phylum has lower average PEC gene counts, and only a subset of Chloroflexi have Fld genes. This latter trend is thought to occur because the Chloroflexi in our tree have highly divergent life strategies, including the mesophilic anaerobic organohalide respirer *Dehalococcoides mccartyi* (Löffler et al., 2013), the aerobic predator *Herpetosiphon aurantiacus* (Kiss et al., 2011), and the aerobic thermophile *Thermomicrobium roseum* (Wu et al., 2009).

### Actinobacteria

These Gram-positive bacteria vary in their PEC specialization, with some having 100% [4Fe-4S] Fd genes (e.g., *Adlercreutzia equolifaciens*) and others having 75% [2Fe-2S] Fd genes (e.g., *Saccharopolyspora erythraea*). Like Chloroflexi, only a subset of the genomes contain Fld genes. These organisms range widely in their total PEC gene counts, with some lacking PEC genes (*Dermacoccus nishinomiyaensis*) and others having 31 PEC genes (*Pseudonocardia dioxanivorans*). This wide variation appears to be the result of a split within the Actinobacteridae class, leaving one half PEC-rich and the other half PEC-scarce. Each half encompasses organisms with many life strategies. Two notable representatives of the PEC-rich organisms include the symbiotic nitrogen-fixing *Frankiaceae* and the antibiotic-producing *Mycobacteriaceae* (Ventura et al., 2007). The PEC-poor half harbors at least six bacterial families (Kineosporiaceae, Dermacoccaceae, Promicromonosporaceae, Actinomycetaceae, Bifidobacteriaceae, Microbacteriaceae) which include many soil bacteria, the gamma-radiation resistant *Kineococcus radiotolerans* (Phillips et al., 2002), and the gastrointestinal-tract inhabiting Bifidobacteriaceae (Ventura et al., 2007). The clustering of PEC-poor and PEC-rich organisms suggests that there has been evolutionary pressure for these trends, but the underlying cause of that pressure is not known.

### Firmicutes

This phylum is split into two major branches: Clostridia and Bacilli. The Clostridia mirrors Archaea in their high total numbers of PEC genes and high [4Fe-4S] Fd abundances. Bacilli, in contrast, contain low total numbers of PEC genes. While different PEC gene types are observed in Bacilli, Flds are most common. In our dataset, Bacilli are largely represented by members of the Lactobacillales order. There are two reasons why these lactic acid bacteria may utilize few Fds. First, most lactic acid bacteria colonize iron-poor environments and have evolved metabolisms that support growth without iron (Duhutrel et al., 2010). Second, many lactic acid bacteria produce H_2_O_2_ in unusually large quantities as part of their metabolism (Imlay, 2019). The harmful Fenton chemistry that can arise from high H_2_O_2_ and iron may have selected for these bacteria to evolve pathways dependent on Flds rather than iron-containing Fds.

### Bacteroidetes

These microbes have diverse PEC gene pools, with total gene counts that vary between zero and twelve. They are unique in possessing organisms that are PEC specialists, with individual members that contain genes encoding only [4Fe-4S] Fds or Flds. The Fld specialists are confined to the Bacteroidales order, which includes the Fld-specialist *Parabacteroides distasonis* and the PEC-generalist *Porphyromonas gingivalis*. Both of these species are commensal and pathogenic bacteria associated with the oral cavity (Naito et al., 2008; Kverka et al., 2011). The [4Fe-4S] Fd specialists include an endosymbiont of amoebas with a reduced genome and severely limited biosynthetic capabilities (*Candidatus Amoebophilus asiaticus*), and an aerobic gliding soil bacterium that digests cellulose (*Cytophaga hutchinsonii*) (Penz et al., 2010; Zhu and McBride, 2017).

### Delta and Epsilon Proteobacteria

These organisms encode [4Fe-4S] Fd genes at higher levels in their genomes compared with the other two PEC types. The sulfur-reducing *Desulfobacula toluolica* is a standout member that exemplifies this trend. This organism possesses twenty-two [4Fe-4S] Fd, five [2Fe-2S] Fd, and four Fld genes. One unusual order in the Deltaproteobacteria class is Myxococcales. These strict aerobic organotrophs metabolize macromolecules like cellulose, and despite having some of the largest bacterial genomes which are 9 to 10 million nucleotides in length, the two Myxococcales species in the pruned phylogenetic tree possess only five PEC genes (Dawid, 2000). In comparison, another myxobactera, *Stigmatella aurantiaca* DW4/3-1, which is not present in the phylogenetic tree, has seven PEC encoding genes (Supplementary Dataset 1). Additionally, this order stands out from the rest of the Delta and Epsilon Proteobacteria because it has predominately [2Fe-2S] Fd genes.

### Alphaproteobacteria

Organisms within this class range from having 0 to 28 PEC genes, with a majority having genomes with at least one [4Fe-4S] Fd and one [2Fe-2S] Fd gene; only half have a Fld gene. Alphaproteobacteria with the highest PEC abundance are in the nitrogen-fixing *Rhizobiales* order (μ = 10.6 ± 7.1). In these organisms, the most abundant PEC genes encode [2Fe-2S] Fds. For instance, *Bradyrhizobium japonicum* has genes encoding nineteen [2Fe-2S] Fds, seven [4Fe-4S] Fds, and two Flds. The two-stage life cycle of Rhizobia, which includes bacteroid and free-living cells, may explain this abundance of PEC genes. In the bacteroid stage, Rhizobia derive reducing equivalents from host roots and, as they are metabolically active but not growing, must often shunt a large portion of those reducing equivalents into lipogenic pathways to maintain redox balance (Terpolilli et al., 2016). This coupling enables nitrogen assimilation, a process which requires Fds for transfer of low potential electrons from central metabolism to nitrogenase (Terpolilli et al., 2016). In contrast, free-living Rhizobia often gain a competitive advantage by metabolizing the secondary products excreted by a bacteroid of the same species, using oxidative pathways that are species-specific and require specialized PECs (Bahar et al., 2000).

One member of the Alphaproteobacteria (*Ensifer adhaerens* OV14) lacks PEC genes. As an opportunistic predator of the rhizosphere, *E. adhaerens* has recently generated interest as an alternative to *Agrobacterium tumefaciens* for the transformation of plants (Rudder et al., 2014). Often living in the nutrient-rich nodules of the rhizosphere, it is thought to depend upon the metabolisms of plants or other rhizosohere microbes to supplement the loss of some PEC-dependent pathways (Rudder et al., 2014).

### Betaproteobacteria

These organisms also have a large variability in PEC gene numbers in each genome, with one organism having 29 PEC genes (*Azoarcus* sp. KH32C) and some containing only three PEC genes (*Nitrosomonas europaea, Candidatus Profftella armatura*, and *Candidatus Kinetoplastibacterium oncopeltii* TCC290E). One interesting order within this class, *Nitrosomonadales*, has genes encoding only [4Fe-4S] Fds. This observation can be contrasted with other Betaproteobacteria genomes, which frequently encode at least one [2Fe-2S] Fd and one Fld. Both of the organisms that are [4Fe-4S] specialists are notable wastewater bioremediators, *Nitrosomonas europaea* and *Nitrospira multiformis*, each of which oxidize ammonium to nitrite (Arp et al., 2002; Norton et al., 2008). How these organisms reduce [4Fe-4S] Fds is an open question. *N. europaea* grows autotrophically on NH_3_, CO_2_, and mineral salts alone (Arp et al., 2002), harvesting reducing power from NH_3_ to produce NADH (Guo et al., 2013) which has a redox potential of−320mV (Huang et al., 2012), well above the potential of many [4Fe-4S] Fds which can range from−280 mV to−650 mV (Atkinson et al., 2016). Measuring the reduction potential of [4Fe-4S] Fds in nitrifying organisms may reveal that they are shifted more positively than canonical Fds.

### Gammaproteobacteria

Organisms in this class are highly balanced in their usage of all three PEC types. This trend may arise because this class contains many pathogens. Endosymbiotic organisms often struggle to scavenge sufficient iron from the host environment, so the substitution of Flds for iron-demanding Fds may have occurred to support fitness under iron-limiting conditions (Litwin and Calderwood, 1993). The *Enterobacteriales* order, which contains pathogens such as *Salmonella enterica* and *Yersinia pestis*, stands out as being highly populated by all three PEC types, presenting a high percentage (up to 50%) of Flds.

### Additional Phyla

Members of Planctomycetes, Verrucomicrobia, Chlamydia, Acidobacteria, and Nitrospirae are represented within the tree, but only in low numbers. In these organisms, [4Fe-4S] Fds are most abundant, although all three PEC types are observed. Additionally, our tree contains small numbers of organisms from the Fusobacteria, Deinococcus, Aquificae, Thermotogae, and Chlorobi phyla. In Fusobacteria, Flds genes are most abundant among the three PEC types, although there are members of this phylum with mixed PEC usage. Many Fusobacteria participate in polymicrobial infections of the respiratory tract and other anaerobic mucosal surfaces (Bennett and Eley, 1993). In these niches, iron availability is predicted to be limited, so Fusobacteria may use Fld genes as part of an evolutionary pressure to be iron frugal. Deinococcus, Aquificae, Thermotogae, and Chlorobi are more dominated by [4Fe-4S] Fds and have mixed PEC usage.

### PEC Usage Varies With Environment Niche

**FIGURE 6 |:**
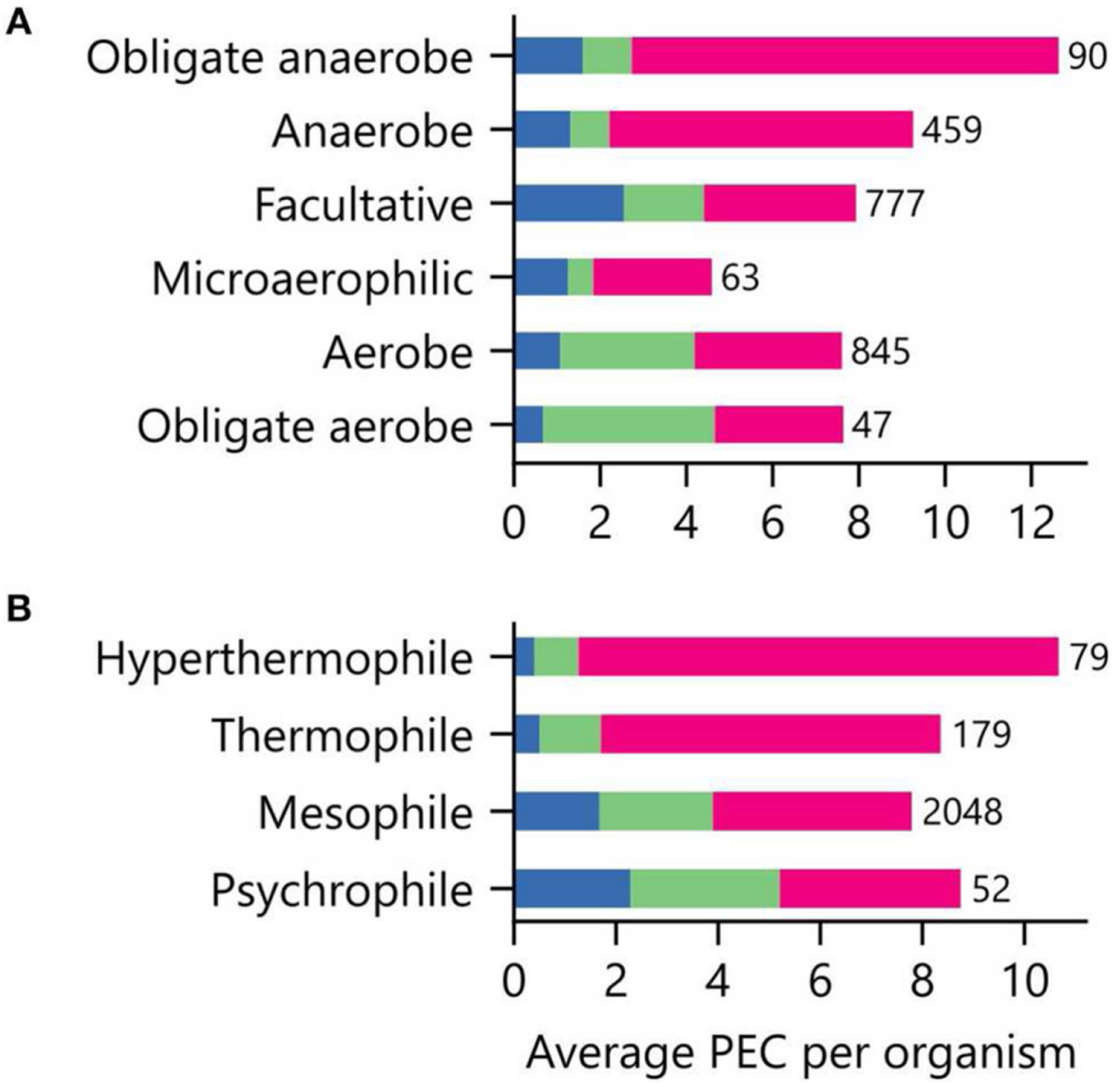
Relationship between environmental niche and PEC use. The average PEC gene counts for organisms having different **(A)** O_2_ requirements and **(B)** growth temperatures. Colors represent the counts of [4Fe-4S] Fd (red), [2Fe-2S] Fd (green), and Fld (blue) gene abundances. The number of genomes of each type is shown adjacent to the bars.

Low potential PECs differ in their iron requirements and stabilities in the presence of atmospheric O_2_ concentrations (Jagannathan et al., 2012; Pierella Karlusich et al., 2014; Holm and Lo, 2016). To determine if an organism’s environmental niche correlates with PEC abundance, we evaluated how an organism’s preferences for O_2_, external pH, and temperature relate to the average number of PEC genes found in a genome. Figure 6A shows that organisms with distinct O_2_ requirements differ in their PEC usage. The [4Fe-4S] Fds, whose cofactors can be damaged by O_2_ (Hsueh et al., 2013), represent the majority of PECs within anaerobes and obligate anaerobes. As O_2_ tolerance increases, Flds and [2Fe-2S] Fds increase in relative abundance, becoming the majority of PECs in aerobes and obligate aerobes. Interestingly, [4Fe-4S] Fd usage is not abolished in aerobes. This observation suggests that some [4Fe-4S] Fds have evolved strategies to protect their [4Fe-4S] clusters from oxidative damage.

Several mechanisms have been proposed to increase [4Fe-4S] stability, including: (1) an elongation that creates a sequence that shields the [4Fe-4S] cluster from O_2_, (2) tight association with partner proteins to achieve [4Fe-4S] shielding (Jagannathan and Golbeck, 2009), and (3) adoption of a [3Fe-4S] cluster as a redox cofactor rather than a [4Fe-4S] cluster (Tilley et al., 2001). Support for the last mechanism has come from successes in purifying Fds with [3Fe-4S] clusters that remain stable under aerobic conditions (Aono et al., 1989; Gomes et al., 1998; Ricagno et al., 2007). Additionally, some [4Fe-4S] clusters lose a single Fe atom when placed in an oxidizing atmosphere, rather than losing the entire cluster (Beinert et al., 1996; Tilley et al., 2001). These findings have led some to posit that Fds that bind one [3Fe-4S] cluster and one [4Fe-4S] cluster (*i.e.*, 7Fe Fds) evolved from ancestral Fds that coordinate two [4Fe-4S] clusters to tolerate O_2_ (Iwasaki et al., 1994; Tilley et al., 2001).

To gain insight into the role that an aerobic atmosphere had on the evolution of 7Fe Fds, we used InterProScan software to analyze the distribution of [3Fe-4S] ferredoxins. For this analysis, we examined the abundance of genes with the IPR signature for 7Fe ferredoxins (IPR000813). This analysis revealed that the [4Fe-4S] Fd genes in obligate aerobes and aerobes matched the 7Fe Fd signature 30 and 27% of the time, respectively (Supplementary Dataset 1). Facultative microbes yielded matches with only 7.8% of the [4Fe-4S] Fd genes, while anaerobes and obligate anaerobes yielded even lower percentages. These trends provide support for the idea that Fds with a [3Fe-4S] metallocluster are more stable under aerobic conditions.

We next investigated how PEC usage relates to growth temperature (Figure 6B). Genes encoding [4Fe-4S] Fds were most prevalent in thermophilic and hyperthermophilic organisms, with abundances of 80% and 88% respectively. These thermotolerant organisms also use [2Fe-2S] Fds and Flds, with the [2Fe-2S] Fds being >2-fold more prevalent than Flds. As optimal growth temperature decreases, the [4Fe-4S] Fds are partially replaced by [2Fe-2S] Fds and Flds.

**FIGURE 7 |:**
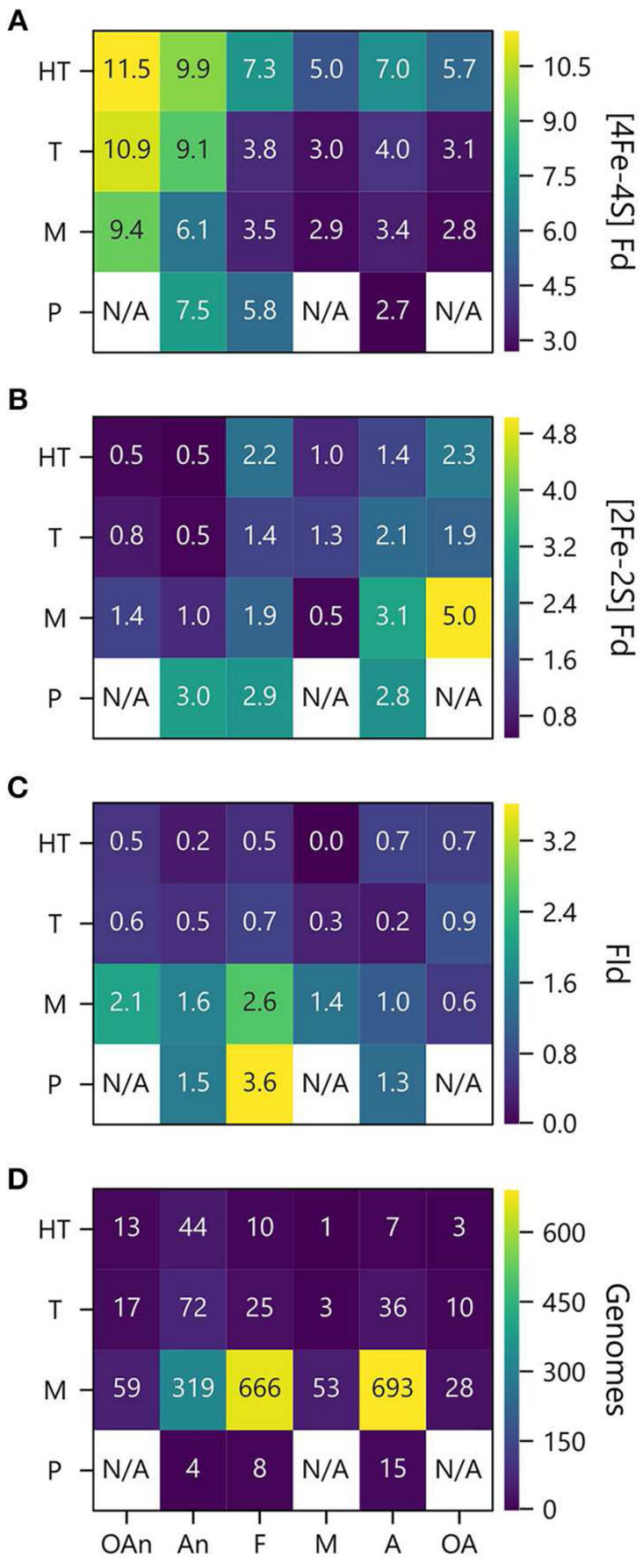
PEC counts sorted by temperature and oxygen niche. The average numbers of **(A)** [4Fe-4S] Fd **(B)**, [2Fe-2S] Fd, and **(C)** Fld gene counts per genome are plotted as a function of O_2_ requirement and optimal growth temperature. **(D)** Genome counts from each environmental niche are plotted as a function of O_2_ requirement and optimal growth temperature. O_2_ requirements shown on the x axis are abbreviated as obligate anaerobe (OAn), anaerobe (An), facultative (F), microaerophilic (M), aerobic (A), and obligate aerobic (OA). Growth temperatures shown on the y axis are abbreviated as hyperthermophile (H), thermophile (T), mesophile (M), and psychrophile (P). N/A denotes not applicable when organisms were not observed.

To investigate how PEC gene abundance relates to both growth temperature and O_2_ preference, we generated heat maps that compare the average number of PEC genes for different combination of conditions. Figure 7A shows that organisms with the greatest abundances of [4Fe-4S] Fd genes live at high temperatures in the absence of O_2_, and those with the lowest abundances live at lower temperatures in the presence of O_2_. Figure 7B illustrates how the organisms with the highest [2Fe-2S] Fd gene counts are mesophiles and obligate aerobes. This analysis also shows that [2Fe-2S] Fds present the lowest abundances at extreme temperatures in the absence of O_2_. Figure 7C shows that Fld genes are most abundant in psychrophiles that are facultative, and this comparison reveals that Flds present similar low abundance across thermotolerant microbes that live in the presence of different O_2_ concentrations. The underlying cause of this trend is not known. However, the paucity of Flds at high temperatures could be caused by the accelerated degradation rates of free FMN cofactor at higher temperatures (Daniel and Cowan, 2000). Figure 7D shows the number of genomes used to analyze PEC abundance in each growth niche category.

**FIGURE 8 |:**
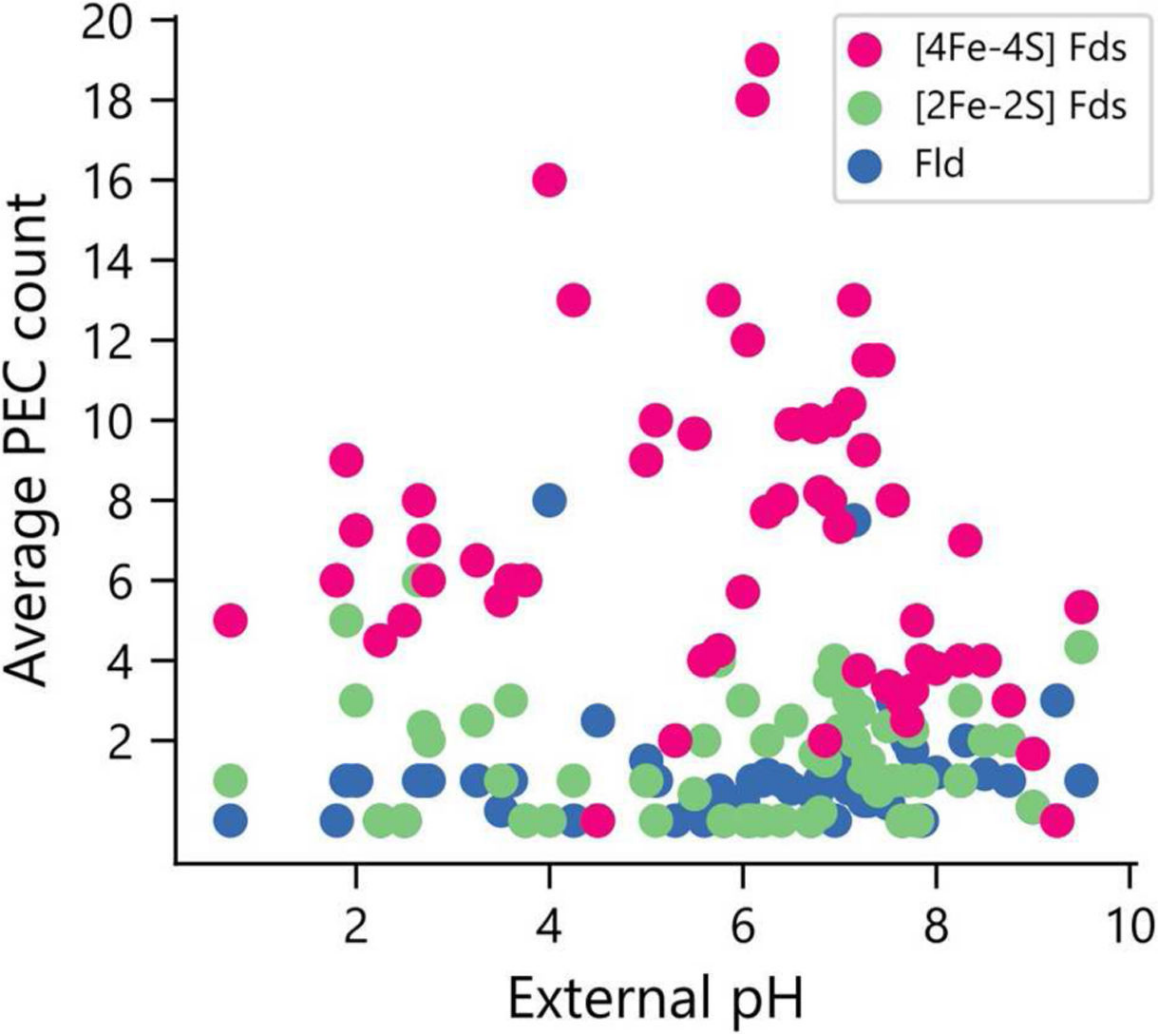
Relationship between environmental pH and PEC gene counts. The distribution of PEC gene counts is normalized by the number of organisms found to grow at each pH value to obtain weighted averages of each PEC type. Analysis of the means revealed no significant pairwise differences using a paired two-tail *t*-test (*p* = 0.126 for [2Fe-2S] Fd and Fld comparison, *p* = 0.163 for [4Fe-4S] Fd and Fld comparison, and *p* = 0.75 for [2Fe-2S] and [4Fe-4S] Fd comparison).

Elevated pH can lead to the formation of iron hydroxides, which can decrease the concentration of accessible iron. To determine if organisms that grow in niches with high pH are enriched in PECs that use organic FMN rather than Fe-S clusters as cofactors, we compared PEC usage in organisms for which data was available on the pH of their ecological niche (*n* = 246). All three PEC types were found in organisms that grow optimally at the extreme ends of the pH scale (Figure 8). The [2Fe-2S] and [4Fe-4S] Fds are found in lower average external pH environments than Flds, although *t*-tests comparing the distributions found no significant difference in their mean values.

### PEC Length Distributions

In organisms with multiple PEC paralogs, studies have revealed that homologs can specialize and evolve partner specificity that allows individual PECs to transfer electrons efficiently to a subset of oxidoreductase partners in an insulated manner (Terauchi et al., 2009). Biochemical studies have shown that this specificity can be achieved by altering the physicochemical properties of the PEC surface to tune binding rates and affinities for specific partners (Akashi et al., 1999), using allosteric conformational changes upon partner binding to regulate ET rates (Tyson et al., 1972; Schlesier et al., 2016), and increasing protein length to create protrusions that sterically hinder binding to some partners (Aoki et al., 1998). With the last mechanism, one would expect that organisms having a single PEC would exhibit smaller average sizes than those family members found in organisms having multiple PECs. To investigate this idea, we sorted PECs by the total numbers of PEC-encoding genes within each genome, and we plotted the distribution of lengths for each PEC type against the total number of PEC genes within genomes.

**FIGURE 9 |:**
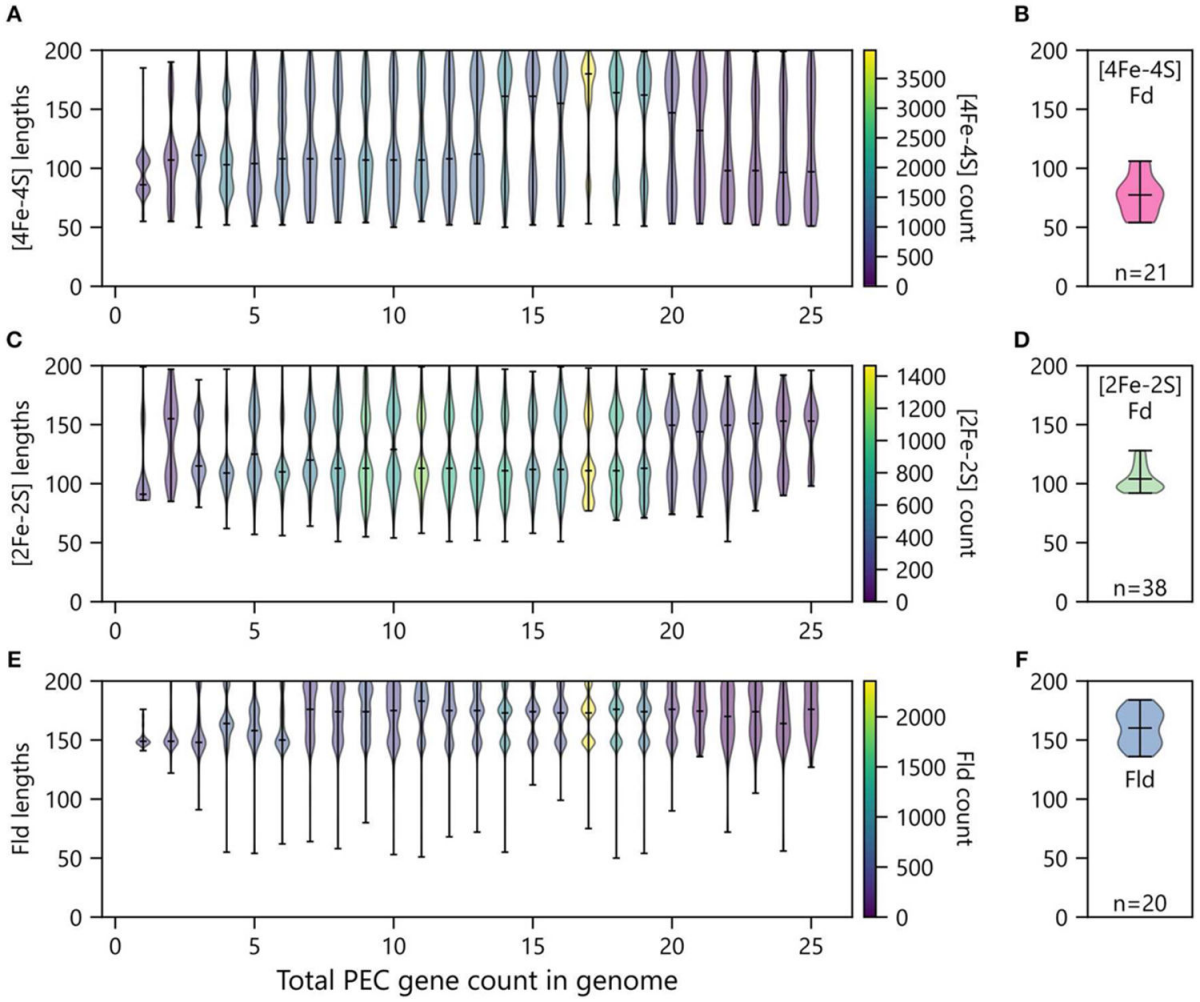
Relationship between PEC length and number of PECs per organism. The abundances of **(A)** [4Fe-4S] Fds, **(C)** [2Fe-2S] Fds, and **(E)** Flds of different lengths encoded by genomes having different *total* numbers of PEC genes. Violin plots show the relative abundance of PECs having different sizes in each bin. The extrema are marked by horizontal edge lines at the ends of the vertical bars, and the average length is marked by an internal horizontal line. The number of genes within each violin plot is visualized using a viridis color gradient. For comparison, the lengths of **(B)** [4Fe-4S] Fd, **(D)** [2Fe-2S] Fd, and **(F)** Fld structures deposited in the Protein Data Bank (Berman, 2000). The number of structures used to generate these plots are noted at the bottom of each panel.

Organisms having only one PEC gene presented smaller [2Fe-2S] and [4Fe-4S] Fds compared with organisms have two or more total PECs (Figure 9). Additionally, organisms with only one PEC gene presented a tighter distribution of Fld lengths compared to organisms possessing multiple PEC genes (Figure 9). For organisms with multiple PEC genes, different numbers of modes are observed for the size of each PEC. For Flds and [2Fe-2S] Fds, organisms having a single PEC display a single mode with average lengths of ~150 and ~90 residues, respectively. These “short-chain” Flds and [2Fe-2S] Fds are well represented in the Protein Data Bank, including *Desulfovibrio vulgaris* Fld (2FX2) (Watt et al., 1991), *Desulfovibrio desulfuricans* Fld (3KAP) (Romero et al., 1996), *Citrobacter braakii* Fld (4OXX) (Madrona et al., 2014), *Trichomonas vaginalis* Fd (1L5P) (Crossnoe et al., 2002), *Equisetum arvense* Fd (1WRI) (Kurisu et al., 2005), and *Scenedesmus fuscus* Fd (1AWD) (Bes et al., 1999). In contrast, organisms having a single [4Fe-4S] Fd display two distinct modes. These modes are centered around ~80 and ~105 residues, respectively. The first mode corresponds in size to di-cluster [4Fe-4S] Fds seen in the PDB and often associated with photosystem I, such as *Synechocystis sp. PCC 6803* Fd (4KT0) (Mazor et al., 2013), *Thauera aromatica* Fd (1RGV) (Unciuleac et al., 2004), and *Thermosynechococcus elongatus* Fd (1JB0) (Jordan et al., 2001). The second mode corresponds to the hybrid [3Fe-4S][4Fe-4S] Fds in the PDB, such as *Sulfolobus tokodaii* Fd (1XER) (Fujii et al., 1996), *Mycobacterium smegmatis* Fd (2V2K) (Ricagno et al., 2007), and *Azotobacter vinelandii* Fd (7FDR) (Schipke et al., 1999).

As the total number of PECs encoded in a genome increases, the average length of each PEC type increases, and the variance around each mode increases. In the case of [4Fe-4S] Fds, multiple distinct modes appear at total PEC abundances greater than two (Figure 9A). These are centered around ~70, ~110, and ~170 residues. The relative abundance of each mode changes with total PEC abundance. At lower total PEC abundances (<10), the two smaller modes dominate and blur into a single continuous mode. As total PEC abundance increases from 10 to 20, the largest mode becomes dominant. At even higher PEC abundances, the lower two modes are most prevalent and appear as a single smear. The [4Fe-4S] Fds in the PDB have a distinct size distribution. Structurally-characterized [4Fe-4S] Fds have lengths ranging from 54 to 106 amino acids (Figure 9B). The protein data bank lacks family members above this length regime, like those in our dataset.

For [2Fe-2S] Fds (Figure 9C), a single mode is observed with organisms that have a single PEC, and two or three modes are observed in organisms having multiple PEC genes. These modes occur at ~90, ~Ί 10, and ~160 residues. The size dispersion of the data around the longer modes is greater than that observed with smallest mode. To date, there have been extensive structural studies of [2Fe-2S] Fds with the shorter lengths, including 22 structures of [2Fe-2S] Fds having <100 amino acids and 16 structures of [2Fe-2S] Fds having lengths ranging from 103 to 128 amino acids (Figure 9D). However, analysis of the size distribution of structurally-characterized [2Fe-2S] Fds reveals that we lack structural information on Fds from the largest mode (~150).

Flds (Figure 9E) clearly present two modes as the number of PECs in an organism exceeds ten, which are centered around 145 and 170 residues. This observation suggests that previously described short and long-chain Flds represent two distinct subtypes that are widespread in nature (López-Llano et al., 2004). In cells with even higher PEC abundances, a third Fld size appears having a mode near 200 amino acids. Conducting BLAST searches on these proteins reveals high similarity to the WrbA family of flavoproteins, which are distinct from short- and long-chain flavodoxins in their capacity to conduct two-electron oxidation and reduction reactions which they utilize in their role as NAD(P)H:quinone oxidoreductases (Patridge and Ferry, 2006; Andrade et al., 2007). While it is clear that traditional Flds and previously studied WbrA proteins differ in their ET roles, we do not know if any natural variants exist that can promiscuously perform both functions. The similar structures of the two protein subfamilies suggests that WbrA homologs may be able to bind to natural Fld oxidoreductase partners, at least transiently. Future biochemical studies will be needed to test this idea. Indeed, analysis of the size distribution of structurally-characterized Flds reveals that only a handful (~10) of each subtype have been studied (Figure 9F).

### Oxidizing Conditions and PEC Size Distribution

**FIGURE 10 |:**
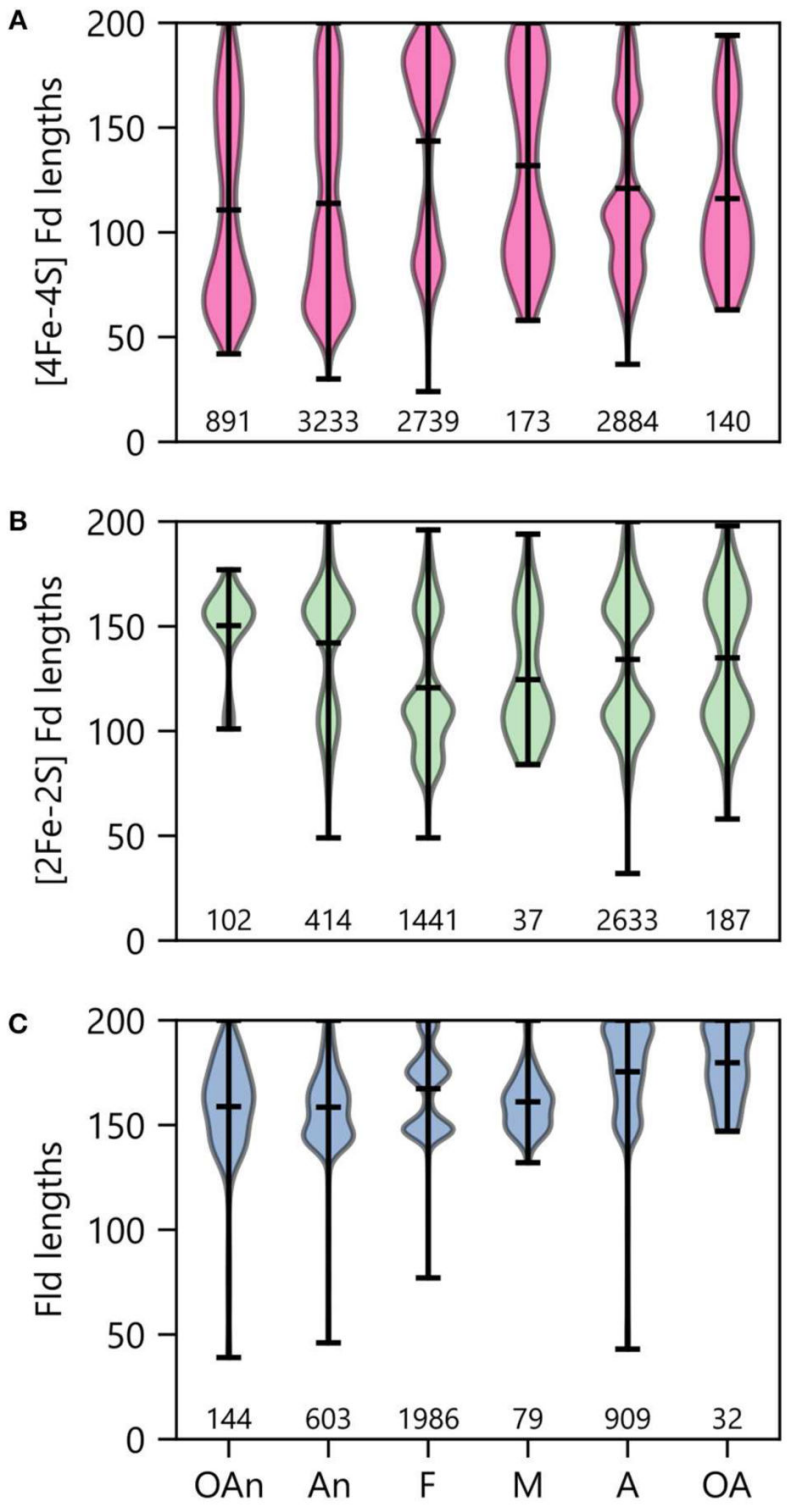
PEC length distributions in organisms having different O_2_ requirements. The lengths of **(A)** [4Fe-4S] Fd, **(B)** [2Fe-2S] Fd, and **(C)** Fld genes in organisms having distinct O_2_ requirements. Growth requirements are abbreviated as obligate anaerobe (OAn), anaerobe (An), facultative (F), microaerophilic (M), aerobic (A), and obligate aerobic (OA). The extrema are marked by the horizontal bars at the edges of the vertical bars, and the average length is noted with an internal horizontal bar. The number of individual genes in each category is listed below each plot.

To probe the relationship between O_2_ growth requirements and PEC lengths within a genome, we binned organisms by ecological niche and evaluated the average size within the modes observed under each condition. With the [4Fe-4S] Fds (Figure 10A), most organisms presented two major modes. The protein length for the larger mode was centered near 170 residues in all cases. However, the average size for the smaller mode increases with the transition from low O_2_ (obligate anaerobes) to high O_2_ (obligate aerobes) conditions. The [4Fe-4S] Fds in aerobes have a length distribution that is devoid of [4Fe-4S] Fds with lengths (≤60 residues) that are characteristic of the prototypical Clostridial Fds (Bertini et al., 1995; Atkinson et al., 2016). This finding suggests that only certain [4Fe-4S] Fds can support ET under the oxidizing conditions where these organisms grow optimally.

When examining [2Fe-2S] Fds length across different O_2_ growth requirements, a distinct trend is observed from the [4Fe-4S] Fds (Figure 10B). The lengths of [2Fe-2S] Fds on the anaerobic end of the spectrum are distributed around a single mode that is centered near 155 residues. In organisms that tolerate and require O_2_, additional modes appear, which are centered at ~80 and 105 amino acids. The underlying cause of this trend is not known. In aerobic organisms, the greater abundance of small [2Fe-2S] Fds may arise to support ET that is challenging for [4Fe-4S] Fds, due to their sensitivity to oxidation.

Interestingly, Flds become longer on average as O_2_ growth requirements increase (Figure 10C). The average Fld length is ~160 residues within obligate anaerobes and ~180 residues in obligate aerobes. In most cases, the Fld lengths are diffusely distributed around their means, with the exception of facultative organisms, which present multiple modes with small variances. There appears to be three Fld lengths that are highly popular in facultative species. Notably, of all the O_2_ requirement groups, we found the most Flds in facultative species (Figure 10C). These organisms are noted for being some of the most enthusiastic Fld adopters with an average percent incorporation of 32% (Figure 6A). Lastly, it is worth noting that in aerobes and obligate aerobes, the Fld length distributions seems to butt up against the ceiling of the 200 amino acid cut-off. This observation suggests that this size cutoff may miss some Flds and that the true length averages for aerobes and obligate aerobes are higher than shown.

### Optimal Growth Temperature and PEC Size Distribution

**FIGURE 11 |:**
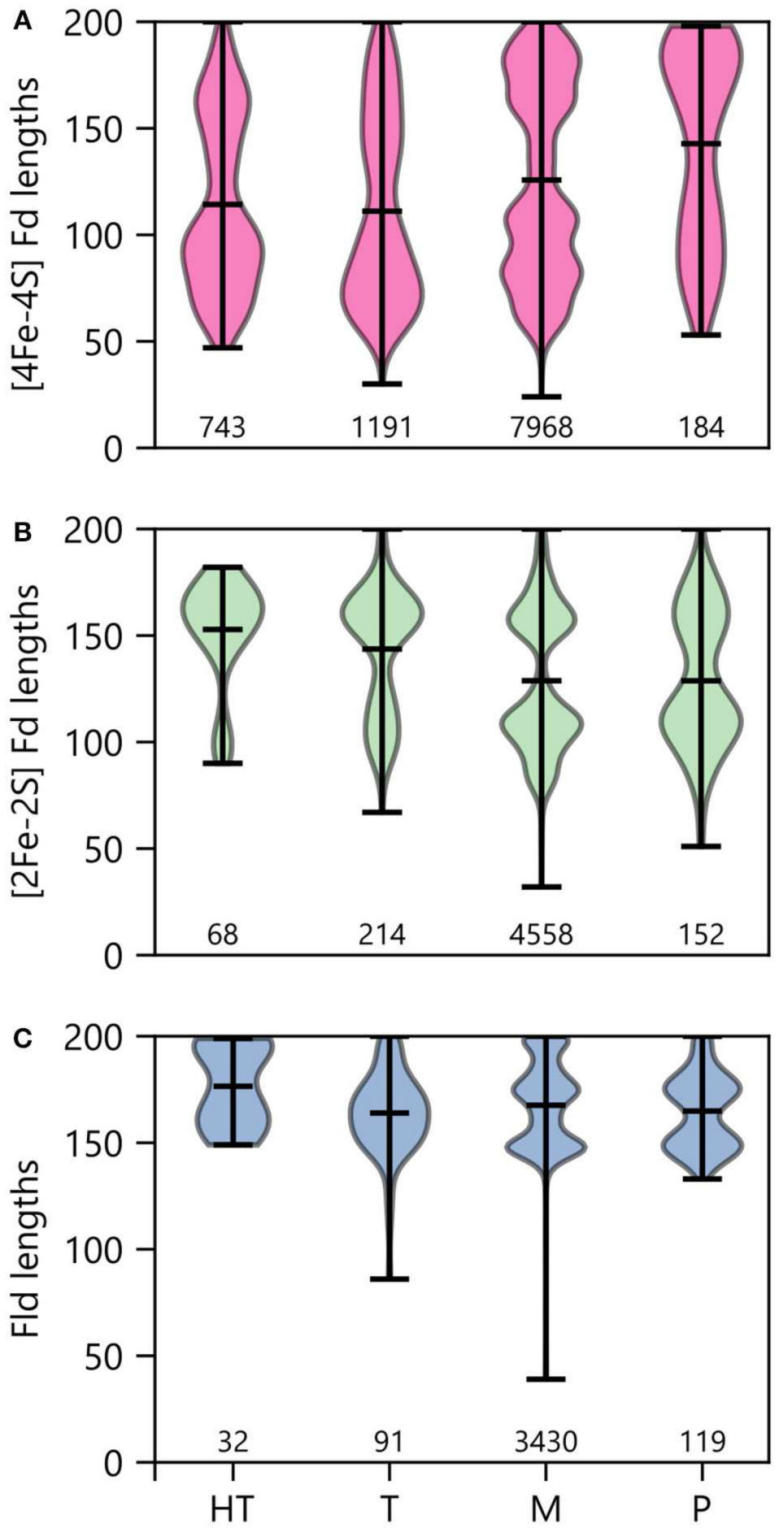
PEC length distributions in organisms having different growth temperatures. The lengths of **(A)** [4Fe-4S] Fds, **(B)** [2Fe-2S] Fds, and **(C)** Flds found in organisms having different optimal growth temperatures is shown using violin plots. Optimal growth temperatures are abbreviated as hyperthermophile (H), thermophile (T), mesophile (M), and psychrophile (P). The extrema are noted with horizontal bars at the edges, and the average is noted with an internal horizontal bar. The number of individual genes in each category is listed below the plots.

To determine how optimal growth temperature relates to PEC gene lengths, we binned PEC types by growth temperature and evaluated the average size under each condition (Figure 11). Under thermophilic and hyperthermophillic conditions, genes encoding [2Fe-2S] Fds and Flds are longer on average than their mesophilic and psychrophilic counterparts. In contrast, there is marked shift toward smaller [4Fe-4S] Fds in thermotolerant organisms. This latter trend is consistent with the proposed emergence of [4Fe-4S] Fds before [2Fe-2S] Fds and Flds in ancient thermophilic archaea (Sousa et al., 2013).

## DISCUSSION

Our bioinformatics analysis reveals that genes encoding low potential PECs are abundant within genomes from organisms across the tree of life. In the 56 phyla studied herein, we found 98% of them harbor at least one organism with a PEC gene. While kingdom level analysis revealed that the average number of PEC genes per organism decreases as one goes from Archaea (~13) to Bacteria (~8) and Eukarya (~3), a large amount of variation was observed within each kingdom. For example, one bacterium contains as many as 54 total small PEC-encoding genes (*Desulfitobacterium hafniense* DCB-2), 53 of which match for [4Fe-4S] PEC genes, and one eukaryote presented 42 PEC genes (*Zea mays*). As one considers the individual protein families analyzed, over 500 organisms presented 10 or more paralogs from either the [4Fe-4S] or [2Fe-2S] Fd families. In contrast only one organism had 10 or more Fld paralogs. The reason why iron-sulfur cluster containing PECs evolved larger numbers of paralogs within extant organisms is not known.

Although many lifeforms maintain an extensive PEC gene pool, suggesting a need for distinct physiological roles for each paralog (Onda et al., 2000; Terauchi et al., 2009; Peden et al., 2013; Cassier-Chauvat and Chauvat, 2014; Atkinson et al., 2016; Mellor et al., 2017; Burkhart et al., 2019), some organisms in our dataset lack annotated genes encoding small (<200 amino acids) PECs. These organisms span over a dozen phyla and all three domains of life. Given the phylogenetic diversity of organisms that lack annotated PEC genes, these organisms are unlikely to share a common metabolism. Further investigation will be needed to determine if these organisms evolved a more extensive use of NAD/NADP-dependent oxidoreductases and/or if they utilize oxidoreductases that arose from the fusion of genes encoding small PECs and their partner oxidoreductase. Such fusion proteins would not have been detected by our 200 amino acid cutoff.

Our size analysis revealed significant variation in PEC lengths. The average lengths of the [2Fe-2S] Fds, [4Fe-4S] Fds, and Flds were smallest in organisms containing a single PEC. This observation suggests that organisms with more than one PEC may require longer primary structures to support increased partner specificity, allowing organisms to discriminate which PEC is involved in an ET pathway (Onda et al., 2000; Terauchi et al., 2009; Duhutrel et al., 2010; Peden et al., 2013; Cassier-Chauvat and Chauvat, 2014; Atkinson et al., 2016; Burkhart et al., 2019). Our size analysis also revealed a large dispersion of gene lengths with multiple modes for each PEC type. For example, multiple modes were observed with the [4Fe-4S] Fds, which varied with the number of total small PECs encoded by genomes. While these modes occurred at different lengths, we observed family members with almost every possible size connecting these modes. This observation can be contrasted with the size distribution of structurally-characterized PECs. A narrower distribution of PEC sizes occurs in the structures within the PDB (Berman, 2000). This finding suggests that one way to obtain greater insight into PEC structural diversity would be to obtain structural data for PECs exhibiting a greater diversity of lengths.

To better understand the underlying reason for variation in PEC lengths, we evaluated how the primary structure of each PEC type changes with organismal growth requirements. We uncovered widespread variation in PEC structure and gene pool makeup that coincides with changes in an organism’s O_2_ requirement and tolerance. Previous research has found that Fd sequence extensions and partner protein binding can both enhance O_2_ tolerance of [4Fe-4S] clusters through shielding (Jagannathan and Golbeck, 2009). In support of this observation, we detected the shortest [4Fe-4S] Fd lengths in anaerobic organisms and a shift toward longer [4Fe-4S] Fds in aerobic organisms, additionally observing that [4Fe-4S] Fds are restricted to fewer permissible lengths under aerobic conditions. Altering the cluster type of a [4Fe-4S] Fd to a [3Fe-4S] Fd by removing one of the Fe atoms has also been hypothesized as a strategy for increasing resistance to oxidative damage (Tilley et al., 2001). In support of this idea, we found that 7Fe Fd gene signatures are more prevalent in aerobic organisms. Furthermore, we observed that [2Fe-2S] Fd and Fld genes make up a larger portion of the gene pool in aerobic organisms, reinforcing the notion that these organisms have been enriched in O_2_-tolerant [2Fe-2S] Fds and Flds. Our findings highlight the need for further *in vitro* studies examining how the O_2_-tolerance of [4Fe-4S] Fds varies with primary structure.

Our bioinformatics analysis supports the idea that [4Fe-4S] Fds represent the most ancient low potential PEC family (Sousa et al., 2013). Organisms harboring [4Fe-4S] Fds were observed extensively across the tree of life, but they occurred with the greatest abundance in Archaea, which are deeply rooted (Sousa et al., 2013). Our pairwise analysis of PEC abundance provides additional evidence for this hypothesis. Organisms with five or more [2Fe-2S] Fds or Flds almost always contain [4Fe-4S] Fds (>99% of the time). However, as the numbers of [4Fe-4S] Fds increases, the abundances of [2Fe-2S] Fds and Flds decrease. Furthermore, it has been theorized that the most ancient organisms on Earth were thermophilic, anaerobic, and similar to modern day Archaea (Sousa et al., 2013). We found that organisms in all three of these categories are enriched in [4Fe-4S] Fds compared with the other PEC types. Taken together, these findings support the theory that [4Fe-4S] Fds enjoyed early adoption across the global microbiome before the Great Oxidation Event and have been maintained in many lineages as [2Fe-2S] Fds and Flds grew in popularity.

One thing that is more challenging to discern is whether [2Fe-2S] Fds or Flds evolved first. Our comparison of the relative abundances of these PECs identified similar numbers of organisms that are [2Fe-2S] Fd and Fld specialists. Additionally, [2Fe-2S] Fds and Flds are both found in a range of organisms that are deeply rooted in the tree of life, including Archaea and Cyanobacteria. However, [2Fe-2S] Fds are most abundant within Cyanobacteria, suggesting that these PECs arose prior to Flds in this phylum to support photosynthesis and diversified through duplication and mutation prior to these organisms evolving Flds. One avenue to unraveling this question may be to examine the PEC “fossil record” from a structural perspective. A recent study using the structural database to study oxidoreductase evolution observed a modular origin of biological ET chains (Raanan et al., 2018). An additional way to elucidate this question is to use protein design to test ideas about ancestral PECs that are no longer observed in nature (Mutter et al., 2019).

In a small number of organisms, proteomic studies have provided evidence that cells differentially control the flow of electrons across metabolic pathways by diversifying their PEC pool. Our finding that PECs are abundant in many genomes across the tree of life illustrates the need to understand the rules that guide PEC partner specificity. Structural studies have provided some insight into the molecular interactions that mediate PEC interactions with partner oxidoreductases, including structures of PEC-partner complexes (Morales et al., 2000; Kurisu et al., 2001; Müller et al., 2001; Dai et al., 2007; Xu et al., 2008; Strushkevich et al., 2011). However, these studies are limited to a small number of protein complexes. Even for the best-characterized PECs, we lack rules for anticipating partner specificities and predicting the electron fluxome. Our understanding of sequence-structure-electrochemical properties further limits our ability to anticipate PEC-mediated control over electron flow in cells. Relatively few PECs have had their midpoint reduction potentials measured (Atkinson et al., 2016), and strategies for characterizing the electrochemical properties of PECs are low throughput due to the need for protein overexpression and purification prior to electrochemical characterization. Unfortunately, algorithms for predicting midpoint potentials from primary structure are not yet sufficiently accurate and robust to predict PEC midpoint potentials without the need for *in vitro* characterization (Perrin and Ichiye, 2013).

In the future, high-throughput methods for comparing the ET efficiencies of PECs with defined partner proteins could help develop rules that relate PEC sequence to partner specificities. Cellular assays that couple biomass production to PEC-mediated ET in synthetic pathways have been reported and utilized to study both natural and synthetic PECs (Barstow et al., 2011; Atkinson et al., 2016, 2019). Such methods could be leveraged to analyze the partner specificities of any PEC imaginable, since genes are cheap to synthesize. We posit that the best PECs to analyze in such assays will be those having divergent sequences, which can be identified using sequence similarity networks (Brown and Babbitt, 2012). It may also be possible to uncover PECs with strong partner interactions by identifying operons that colocalize PECs with their partner oxidoreductases (Gerlt, 2017). Further biochemical studies will be required to evaluate whether oxidoreductases colocalized with PECs exhibit greater specificity for one another compared with PECs encoded in more distal genomic regions. We hypothesize that the quickest way to obtain this information will be through high-throughput cellular assays that couple electron transfer between a PEC and its partner to cell growth (Atkinson et al., 2016). Large amounts of specificity data can be generated by expressing different PEC homologs in the presence of the same partner oxidoreductases and cataloging differences in growth that are observed with different PEC-partner combinations (Barstow et al., 2011; Atkinson et al., 2019). Since growth is proportional to electron transfer to a partner protein, the growth data obtained in such assays reflects the relative specificity of PECs for the same partner protein.

## Supplementary Material

Supplementary Dataset 1

## Figures and Tables

**TABLE 1 |:** The average PEC gene counts for each phylum is displayed, including the number of [4Fe-4S] Fd, [2Fe-2S] Fd, and Fld genes, as well as the sum of all three types.

Phyla	< Protein electron carriers >			Total	Genomes
	[4Fe-4S]	[2Fe-2S]	Fld		
Streptophyta	6.50	18.50	2.00	27.00	2
Euryarchaeota	11.91	1.13	1.19	14.23	190
Deltaproteobacteria	10.71	1.77	0.76	13.24	79
Acidithiobacillia	7.50	4.50	1.00	13.00	4
Cyanobacteria	4.35	7.40	1.00	12.75	109
Gammaproteobacteria	5.75	2.62	4.18	12.55	2091
Synergistetes	7.22	3.11	0.67	11.00	9
Betaproteobacteria	3.87	5.62	1.05	10.54	557
Crenarchaeota	8.02	1.73	0.47	10.22	66
Candidatus Korarchaeota	10.00	0.00	0.00	10.00	1
Deferribacteres	8.80	0.20	1.00	10.00	5
Calditrichaeota	8.00	2.00	0.00	10.00	1
Nitrospirae	7.22	1.89	0.22	9.33	9
Chloroflexi	6.63	1.69	0.81	9.13	32
Caldiserica	6.00	3.00	0.00	9.00	1
Ignavibacteriae	7.50	1.00	0.50	9.00	2
Bacillariophyta	2.00	5.00	2.00	9.00	1
Lentisphaerae	6.33	1.67	1.00	9.00	3
Thermodesulfobacteria	8.75	0.00	0.00	8.75	4
Chlorophyta	1.50	6.00	1.00	8.50	2
Thermotogae	6.13	1.10	1.03	8.26	31
Alphaproteobacteria	3.47	3.90	0.66	8.03	566
Chlorobi	6.33	0.40	0.80	7.53	15
Acidobacteria	3.09	3.82	0.36	7.27	11
Chrysiogenetes	7.00	0.00	0.00	7.00	1
Dictyoglomi	4.50	0.00	2.50	7.00	2
Gemmatimonadetes	2.33	4.33	0.00	6.66	3
Aquificae	5.13	1.07	0.20	6.40	15
Thaumarchaeota	5.87	0.00	0.33	6.20	15
Oligoflexia	2.00	3.00	0.43	5.43	7
Fibrobacteres	4.40	0.00	0.80	5.20	5
Planctomycetes	3.35	1.06	0.65	5.06	17
Epsilonproteobacteria	3.90	0.06	1.07	5.03	272
Candidatus Cloacimonetes	3.50	1.50	0.00	5.00	2
Actinobacteria	2.91	1.61	0.41	4.93	700
Deinococcus-Thermus	2.88	1.33	0.46	4.67	24
Armatimonadetes	2.60	1.80	0.20	4.60	5
Fusobacteria	0.85	0.19	3.54	4.58	26
Chordata	0.50	4.00	0.00	4.50	2
Firmicutes	2.14	0.67	1.55	4.36	1401
Elusimicrobia	3.67	0.00	0.33	4.00	3
Bacteroidetes	2.16	1.28	0.48	3.92	248
Verrucomicrobia	2.38	0.50	0.56	3.44	16
unclassified	1.29	2.00	0.14	3.43	7
Spirochaetes	1.19	0.76	0.62	2.57	149
Apicomplexa	0.00	1.83	0.00	1.83	12
Ascomycota	0.03	0.95	0.75	1.73	40
Chlamydiae	0.03	1.03	0.00	1.06	148
Nanoarchaeota	1.00	0.00	0.00	1.00	2
Candidatus Micrarchaeota	1.00	0.00	0.00	1.00	1
Microsporidia	0.00	1.00	0.00	1.00	4
Nematoda	0.00	1.00	0.00	1.00	1
Basidiomycota	0.00	0.67	0.00	0.67	3
Tenericutes	0.18	0.02	0.12	0.32	155
Candidatus Saccharibacteria	0.00	0.00	0.00	0.00	2

The average PEC gene counts for each phylum is displayed, including the number of [4Fe-4S] Fd, [2Fe-2S] Fd, Fld genes, as well as the sum of all three types. Phyla are listed in descending order of total average PEC genes.
